# A flow virometry process proposed for detection of SARS-CoV-2 and large-scale screening of COVID-19 cases

**DOI:** 10.2217/fvl-2020-0141

**Published:** 2020-08-18

**Authors:** Niraja Soni, Puja Pai, Gurumurthy Raja Krishna Kumar, Venkatesh Prasad, Santanu Dasgupta, Bhaskar Bhadra

**Affiliations:** ^1^Synthetic Biology Group, Reliance Corporate Park, Reliance Industries Ltd, Ghansoli, Navi Mumbai 400701, India

**Keywords:** COVID-19, flow cytometry, flow virometry, SARS, SAR-CoV-2, severe acute respiratory syndrome

## Abstract

The viral pneumonia COVID-19, caused by the severe acute respiratory syndrome coronavirus 2 (SARS-CoV-2), has spread rapidly over 210 countries and declared as pandemic by WHO. WHO has emphasized on the scale-up of testing capacity, followed by isolation of infected individuals, and contact tracing, as the ‘backbone’ of managing the pandemic. Globally, the detection of SARS-CoV-2 in patients is done by real-time PCR (RT-PCR) and blood antibody-based testing. Here, a flow cytometry-based high-throughput screening system is proposed for testing of COVID-19 cases where the virus particle binds to specific primary antibodies and the resultant virus–antibody complex then binds to fluorescent-tagged secondary antibodies. The fluorescence signal could be measured in a flow channel for qualitative detection of virus in the test sample.

Flow cytometry is a laser-based cell biology technique that is used to analyze, count and sort cells of interest from a mixed population. Fluorescence-activated cell sorting (FACS) is a method of choice for analysis and purification of isolated single cells (viz., bacteria, algae, plant and animal cells). FACS can detect and discriminate cells as well as suspended particles by its properties of light scattering and fluorescence (excitation/emission mode). The fluorescence of cells may be obtained using specific fluorochrome reagents, or by using antibodies tagged with a fluorochrome targeted against a cell surface antigen and/or internal constituents in permeabilized cells. Flow cytometry has been used for monitoring cells expressing fluorescent proteins (e.g., GFP) [1] and undergoing DNA replication and cell cycle as well as apoptosis. The tool has also been used for immunophenotyping. FACS has been successfully used for generating qualitative and quantitative data in a broad range of biomedical, clinical and therapeutic research, thereby widening its applications from research to clinical studies. The analysis of viruses by flow cytometry was termed as ‘flow virometry’ [2,3].

The recent coronavirus pandemic COVID-2019 is caused by the severe acute respiratory syndrome coronavirus 2 (SARS-CoV-2). The virus belongs to the Sarbecovirus subgenus (genus *Betacoronavirus*, family Coronaviridae). This virus has unique clinical characteristics and is highly contagious with unclear pathological mechanisms. The virus can cause a life-threatening respiratory illness in humans, especially to the people in their late 50s and above, with or without co-morbidities such as diabetes, kidney diseases, heart diseases, etc. Early symptoms of SARS-CoV-2 infection are very similar to that of mild-to-moderate flu, which makes it extremely difficult to classify and shortlisting infected individuals. In addition, inefficient contact tracing of infected individuals is becoming increasingly hard.

Until a successful treatment strategy is appropriately identified, the key to managing this pandemic is greatly dependent on quick and faster detection of infected individuals, followed by isolation of patients from the healthy population. Presently, the detection of the COVID-19 is done by quantitative real-time PCR (qRT-PCR) using unique set of PCR primers. The test detects the viral RNA in the samples, and the first step of the process is making cDNA from viral RNA using the reverse transcriptase enzyme. Immunological detection of SARS-CoV-2 antibodies in the blood of infected humans is another method being used for development of rapid detection kits using lateral flow immunochemistry. Reverse transcriptase – LAMP (loop-mediated isothermal amplification) is another potential detection system for SARS-CoV-2, which uses isothermal amplification of the viral nucleic acid using specially designed oligo-nucleotide primers. Several PCR-based high-throughput diagnostics kits for detection of SARS-CoV-2 are also under development [4]. qRT-PCR, despite being a gold standard and a widely used tool, is time consuming and the throughput of the system is low. The antibody-based detection is not accurate and is prone to false or nonspecific results. Therefore, to cover the larger population, we need an accurate, high-throughput and faster sample testing tool for screening. India is among the top three countries with respect to the number of COVID-19 cases and over the last 2 months the rate of infection has grown exponentially. At present, India is testing close to nine thousand people per million of population, which is clearly insufficient, and therefore a large-scale and robust screening process needs to be developed.

Recently, a human monoclonal antibody that binds to a conserved receptor-binding domain on the spike of the SARS-CoV-2 has been reported [5]. Such monoclonal antibodies against SARS-CoV-2 will be useful for development of antigen detection tests and serological assays. Döhla *et al.* reported 88.9% specificity in qRT-PCR, whereas 36.4% specificity was reported in antibody-based rapid detection kits for diagnosis of COVID-19 cases [5]. Here, we recommend an approach for diagnosis of SARS-CoV-2 by screening of test samples (swabs) using flow cytometry. In this approach, we have proposed a process of indirect immunofluorescence where the virus particles are first bound to primary antibodies followed by the complex being labeled by fluorescent secondary antibodies for detection in a flow cell.

## Materials & methods

### Flow virometry

Flow cytometer was used to detect 70 × 200 nm long T2 phages fixed with glutaraldehyde or formaldehyde [6]. Characterization of viruses using flow cytometry was pioneered decades ago. Advanced flow virometry has now been used to characterize several viruses such as lambda phage, HIV, HSV-1, mouse hepatitis virus, vaccinia virus, dengue virus (DENV), Junin virus, human cytomegalovirus, Nipah virus and giant viruses. Fixing, labeling of the viral particles, careful sample preparation and optimized heating to promote the penetrance of the dye in the virion are the most important steps. For characterization, sorting and study of viruses, flow virometry is emerging as a powerful tool for future [1].

### Flow cytometry to study viruses

Virus particles can be detected in a flow cytometer either based on fluorescence or on the size of the particle. There are many examples where virus particles of various shapes and sizes were sorted or detected using advanced flow cytometry methods (Table 1). Labeling of viral capsid using fluorescent lipophilic dye, labeling genetic materials (DNA/RNA) using nucleic acid binding dye, and labeling with fluorescent immunoglobulin tagged magnetic nanoparticles (MNPs) are few of the widely used methods for detection of virus particles. These are described below.

**Table 1.** Labeling and detection of viruses of different sizes using flow virometry.

Virus	Diameter (nm)	Labeling and dye	Application	Ref.
HIV-1 virion	120–150	Label-enveloped viruses – lipid dyes (DiO, DiD)	HLA DR/LFA1 heterogeneity	[2]
HSV-1 virion	125	Nucleic acid stain – Syto 13	Sorting of nuclear capsid	[2]
HSV-1 virion	250	GFP tagging to diverse virion components	Variability of Tegument and viral fitness studies	[7]
HIV-1 virion	120–150	MNPs	Heterogeneity of HLA DR/LFA1 Studies on envelope	[8]
Dengue virus/virion	40–60	Fluorescent lipidic dye – DiI, fluorescently labeled 2H2 anti-prM antibodies, MNPs	Studies virion maturation	[3,9]
T4 (bacteriophages)	70 × 200	Nucleic acid stain – SYBR green I	Genomics of phages	[10]

DiD: 1,1′-Dioctadecyl-3,3,3′,3′-tetramethylindodicarbocyanine 4-chlorobenzenesulfonate salt; DiO: 3,3′-Dioctadecyloxacarbocyanine perchlorate; DiI: 1,1′-Dioctadecyl-3,3,3′,3′-tetramethylindocarbocyanine perchlorate (’DiI’; DiIC18(3)))); MNP: Magnetic nanoparticle; YOYO™: 1-Iodide {1,1′-(4,4,8,8-tetramethyl-4,8-diazaundecamethylene)bis[4-[(3-methylbenzo-1,3-oxazol-2-yl)methylidene]-l,4-dihydroquinolinium] tetraiodide}.

### Forward light scatter & reduced wide angle for size

Virions (virus particles) show great variation in sizes ranging from 15 nm for nonenveloped circovirus [11] to 350 nm for larger-enveloped vaccinia viruses [12], and up to 1 μm giant viruses [13,14]. In standard flow cytometers, detectors of forward light scatter (FSC) depict the size of a cell or a particle passing through the flow channel. The small-sized viruses when analyzed on flow cytometer fall in the range corresponding to optical, electrical and filtered sheath buffer background noise. Typically, FSC detectors monitor light in the 0.5–15° range; most of the background signals are found in this range. Later, the concept of ‘reduced wide-angle FSC’ detection, which blocks light in the 0–15° range to reduce noise and monitor light at angles between 15 and 70°, was demonstrated [1]. This has greatly improved the signal-to-noise ratios. Impurities from signal could further be reduced by filtering the sheath buffer with a 0.1-μm filter, instead of a 0.22-μm filter which is routinely used. This is highly recommended for the study of smaller-sized virions. In addition, filtering test samples with a 0.45-μm pore, when possible, will be very useful to reduce aggregates and artefacts during analysis [7,8].

### Labeling genetic content

Labeling the genetic content of viruses is another highly, and perhaps, the most efficient approach to detect virions in samples using flow cytometry. The viral nucleic acid is enclosed inside the protein capsid, and the capsid is often covered with one or several distinct layers of proteins that is known as matrix or tegument layer. Almost all enveloped viruses contain a lipid bilayer very similar to host cell membrane. The lipid bilayer and capsid inhibit the penetration of acid dyes; therefore, only few selected dyes have been able to stain the viral DNA/RNA. The examples of nucleic acid dyes used for labeling viral DNA/RNA include YOYO-1, SYBR green-I, PicoGreen and TOTO-1 [15–18]. However, there are reports suggesting that SyBR green-I does not always efficiently label viral particles unless they are first heated to a temperature of 80–90°C [15,18]. After screening several commercial dyes, the membrane-permeable Syto 13 (green fluorescence) or Syto 61 (red fluorescence) worked best for labeling HSV-1. Both these dyes resulted in good signals with very low background noise and excellent sample penetrance [7,8]. Most importantly, the Syto dyes are not damaging to the virion membrane at the concentration of 1 μM that was used and therefore a plaque assay could be done for confirmation.

### Labeling with fluorescent lipophilic markers in the case of enveloped viruses

Lipid dyes are also being used for labeling of enveloped viruses for flow cytometry analysis. This approach was successfully employed for DENV, vaccinia virus and HIV, etc. DiO, DiD and DiI were used for labeling [2,9,19]. One of the most commonly used dyes for labeling enveloped (animal) viruses is PKH67 (green fluorescent cell linker), which is a green fluorescent molecule with an aliphatic tail that targets lipid bilayers [20,21]. SARS-CoV-2 has a lipid bilayer and therefore, PKH67 could potentially be used for labeling and screening of COVID-19 cases.

### Labeling with antibodies coupled to fluorescent moiety

Labeling virus particles with primary antibodies (IgG/IgM with a fluorescent tag) specific to capsid proteins is another approach, which could be used successfully for screening. In case fluorescent tagging of the primary antibody is not possible, a fluorochrome-tagged secondary antibodies which target primary antibodies (IgG/IgM) could be used. To improve detection of the virus, in some cases, the antibodies have been prebound to nanobeads (15 nm), which are also known to enhance light scattering [2,3] during FACS analysis.

### Labeling with MNPs tagged with fluorescent immunoglobulins

Qualitative and quantitative virometry analyses in flow cytometer were successfully done via application of MNPs. The technology is based on binding of the virus molecules to the magnetic beads. The magnetic beads are incubated with a virion-specific antibody. The ‘MNP–antibody’ complex is then mixed and incubated with the virions for immobilization. The immobilized ‘MNP–antibody–virion’ complex is then incubated with secondary antibodies with fluorescent tags. Later, magnetic bound virion complex is separated using a magnetic column and analyzed in a FACS system. The use of MNPs has been reported in the study of HLA DR/LFA1 heterogeneity of HIV-1 [2].

Apart from enumeration of viruses, flow cytometry is used in detection of viral load in peripheral blood mononuclear cells (PBMCs). Viral infections result in a range of immune responses in the body and PBMCs are studied to score the level of infection. Flow cytometry could be used to analyze PBMCs, and FACS is employed to sort each subpopulation of cells. Similar studies were done for lymphocyte subpopulation distribution in the samples of COVID-19-infected patients, and thereafter, compared the data with the cases where SARS-CoV-2 was not the cause of pneumonia [22].

To better understand and facilitate discovery in the immune response to COVID-19, comprehensive research tools are made available. It includes solutions for immunophenotypic, transcriptional and functional analysis of immune cells that cover major areas like viral immune response, cytokine analysis, vaccine research, biomarkers and therapeutics (BD Biosciences).

With the above understanding, we propose the detection of SARS-CoV-2 in the samples using antibodies coupled to a fluorescent moiety.

### Process & method of COVID-19 detection

In this communication, we have discussed a process of advanced flow virometry to enhance testing scale of COVID-19 cases. SARS-CoV-2 belongs to the family *Coronaviridae*, consisting of a 29–30 kb chain of positive single-stranded RNA. The virus particle size ranges from 70 to 90 nm [23]. Extensive studies on the dengue virion (40–60 nm) was done using combination of fluorescently labeled antibodies and MNPs [9]. Here, we propose the approach of labeling the surface of the viral particles with antigen-specific primary antibodies and secondary antibody conjugated to a fluorescent dye (e.g., fluorescein isothiocyanate, PE, Cy5^®^, etc.) to detect the SARS-CoV-2. Proposed steps for sample preparation and assay are discussed in following points (a–h). However, there are potential risks of handling the live SARS-CoV-2 particles in the flow cytometer. Therefore, the process requires a biosafety level 3 (BSL-3) facility and a negative pressure enclosed chamber or a specially designed biohood for reduction of chances of infection caused by potential microdroplets. The steps are:

(a) Collection of oral/nasal swabs in tubes containing viral transport media, and filtering samples with a 0.45-μm cut-off membrane, which is required to minimize aggregation and artifacts in the sample.
(b) Suspension of the samples in filter-sterilized ice-cold phosphate-buffered saline (PBS) solution is the first step of sample preparation. Reports suggest that 1% w/v sodium azide in ice-cold PBS helps to prevent the modulation and internalization of surface antigens, which can help in detection process by improving fluorescence intensity of virion particles.
(c) In 1 ml of the PBS suspension, 0.1–10 μg of the primary antibodies is added. The suspension is mixed, and the tube is incubated in dark for 30–60 min at room temperature. The tube could also be incubated for longer time at 4°C in the dark. Dilutions, if necessary, should be made in solution containing 3% (w/v) bovine serum albumin (BSA) in ice-cold PBS.
(d) After incubation, the washing step is done for three-times by centrifugation at 400 × *g* for 5 min at 4°C. The pellet is resuspended in ice-cold PBS by gentle tapping (vigorous vortexing may reduce efficiency in detection step).
(e) Dilution of the fluorochrome-labeled secondary antibodies could be done in 3% w/v BSA in ice-cold PBS (or according to the manufacturer’s instructions). In 1 ml of the suspended virion-antibody mix from the previous step, 0.2–10 μg of secondary antibodies is added, and the tubes are incubated in dark for at least 30 min at room temperature.
(f) The cells are to be washed three-times by centrifugation at 400 × *g* for 5 min using 1 ml of ice-cold PBS containing 3% (w/v) BSA, 1% (w/v) sodium azide. The supernatant is removed using micropipette and the pellet is suspended in 100–200 μl of ice-cold PBS.
(g) Analysis of the cells on the flow cytometer should be done as soon as possible. We recommend that for virus studies, filtration of the sheath with 0.1-μm filter instead of 0.22-μm filter paper. Viruses are small, therefore, proper thresholds needs to be set for forward side scatter (FSC) and side scatter (SSC). For example, for T4/lambda particle (70 × 200 nm) FSC photomultiplier tubes (PMT) was set at 1000 and SSC at 200 to maximize signal-to-noise ratios. We propose to optimize FSC and SSC (1000 and 400) for enumeration of SARS-CoV-2.
(h) Controls: prior to sample analysis, a blank, in other words, filtered PBS, needs to be analyzed for background event recognition. The analysis needs to be done at low flow rate and readings should be captured on biexponential plots for fluorescence signals (linear scale for FSC and SCC). Surface labeling with primary antibodies and the antigen–primary antibodies–secondary antibodies interaction may not be strong enough if sample processing is not done carefully. Poor sample processing may result in shedding of labeled antibodies from viral surface, which could give false-negative results. Therefore, a viral positive control with known fluorescent intensities should be used as internal control for large-scale analysis.

Flow cytometry could detect DENV after 24 h postinfection in Vero 76 (African Green monkey kidney) cell line [24]. The detection was made possible using fluorescein isothiocyanate-labeled 4G2 monoclonal antibody [25]. Therefore, we propose that early detection of SARS-CoV-2 in suspected patients is possible in flow virometry. Samples with lower viral load may still work better for qRT-PCR-based detection, whereas for the samples with moderate to high viral load, the flow virometry method discussed here will work at an acceptable range of reliability and reproducibility. However, the antigen–antibody-based fluorometric detection, we describe here requires further validation and comparison with other test methods at a larger-scale across various countries.

## Results & discussion

**Figure 1.**
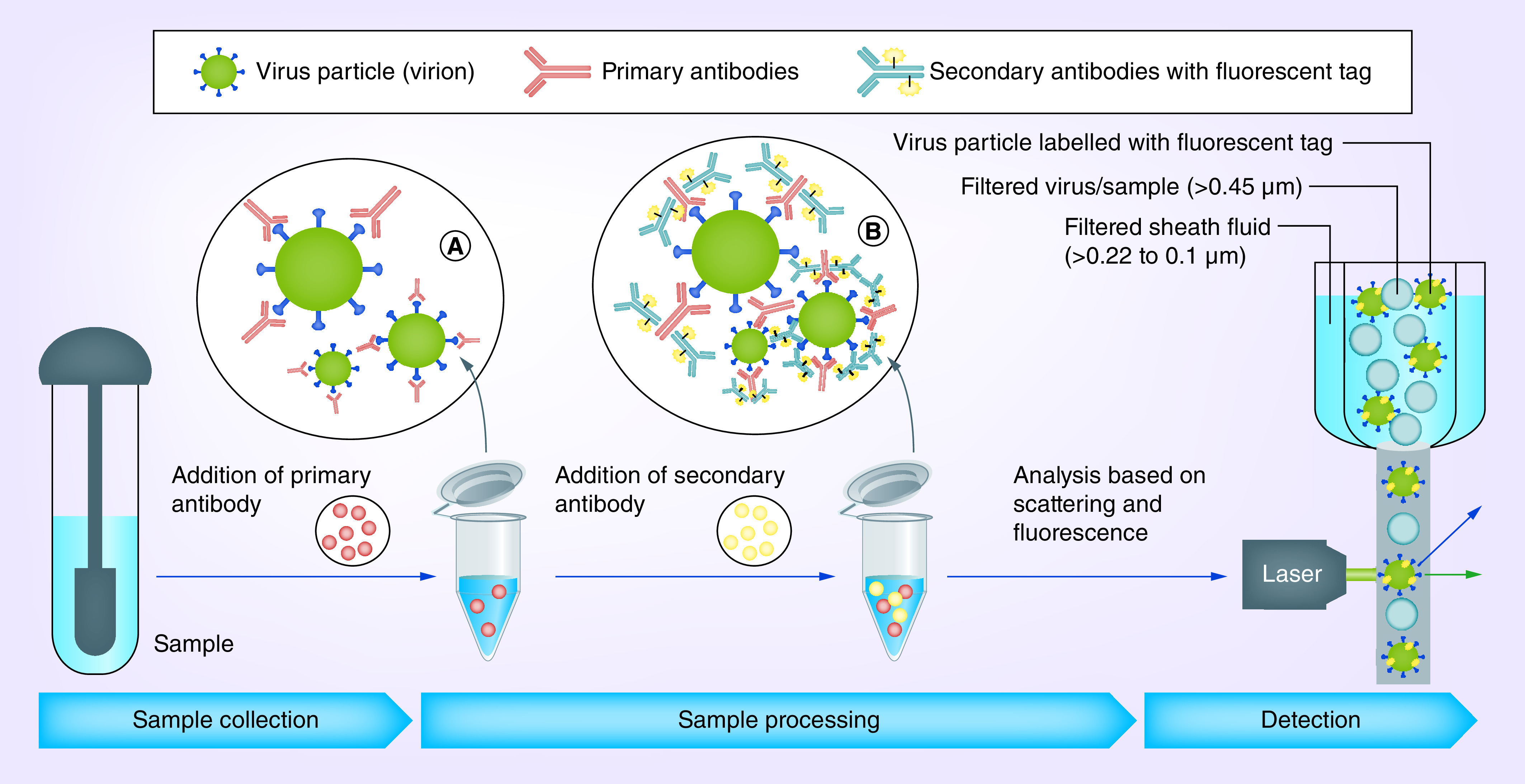
The flow diagram showing steps in sample preparation and detection of the severe acute respiratory syndrome coronavirus-2 using flow cytometer. Test samples in viral transport media mixed with primary antibodies and then with secondary antibodies labeled with fluorescent tag. Samples were analyzed in flow cytometer using laser. **(A)** Binding of viral surface proteins with primary antibodies. **(B)** Fluorescent tagging of virus–primary antibody complex with secondary antibodies.

Our hypothesis of screening COVID-19 samples using flow virometry could be tested in all hospitals, institutes and diagnostic centers equipped with BSL-3 facilities. We also strongly recommend the researchers to follow the personal protective equipment guidelines to test this method in a dedicated instrument. The indirect immunofluorescence protocol discussed in this article where sequential binding of virus particles with primary and fluorescent-tagged secondary antibodies (specific binding to primary antibodies) would give sensitive and faster detection of SARS-CoV-2 in test samples. Outline of process-flow is depicted in Figure 1, where the test sample is incubated with the primary antibodies against SARS-CoV-2, followed by secondary antibodies tagged with a fluorochrome. Similar methods were applied for detection of other pathogenic viruses such as DENV at 3 × 10^5^ particles per ml of culture [25]. Using 18-color SORP sorter (BD FACSAria II) with 355–640 nm lasers, 1.0 FSC ND filter and PMT – SSC detector, flow cytometry could detect 80 virion/ml [26]. The primary antibody needs to be specific and can be one from human anti-SARS-CoV-2 S1 or human anti-SARS-CoV-2 spike receptor-binding domain. To study the limit of detection of an assay setup, titration of the antibodies is required against known concentration of virion. In BD FACSAria II, SSC detector could be positioned at the right angle to the stream and FSC around 10−5° angle will work best for labeled virus particles.

Antigen selection is the most crucial step in order to distinguish SARS-CoV-2 from other coronaviruses. Immune-informatics studies [27] identified an epitope, ITLCFTLKR, which has not only higher binding scores but also 99.8% structural favorability as per Ramachandran-plot analysis root-mean-square deviation (RMSD; values: 0–1 Å). More than 20 epitopes were proposed, and biological stability of the peptides were scored after detailed molecular dynamics simulation and *in silico* codon adaptation experiments [28]. Process optimization and comparative studies with a bunch of epitopes will help in identifying the top three epitopes, which will work best for the method described here.

Overall process of labeling the virion with the antibodies is expected to take around 90–120 min, and sample analysis in flow cytometer takes around 30 s. In a 96-well plate processing of 85–90 samples (including controls) could be done in 180 min in manual operations. However, the whole process should be done in lesser time if a robotic liquid handler is added in the processing step. Therefore, in a robot-assisted screening platform, 1500–1800 samples could be screened in a day using single FACS instrument in a cost-effective manner. Pooled sample analysis for scoring community spread studies will help to scale-up the analysis by ten- to 20-times per day based on design of the experiment. Overall objective to add robotic automated and liquid handler in the process is to minimize human interaction and achieve throughput.

## Conclusion & future perspective

As there are no approved drugs for treatment yet, COVID-19 has now become a major challenge all over the world. Several plant-derived alkaloids such as chloroquine, hydroxychloroquine, bidebiline E, bisnordihydrotoxiferine, thalifaberine, etc., are being studies for combating this virus [29]. Studies across the globe over the last few months clearly show that the SARS-CoV-2 is highly contagious, transmitted by asymptomatic patients/individuals and the infection can be extremely severe for some individuals, which requires patients to be hospitalized with treatment in intensive care units. To detect the virus circulating within local communities, a quick, sensitive and accurate detection of the infection is highly desirable. The process discussed in this article will not only help in quick and high-throughput detection of infection in densely populated habitats but will also help in reducing dependencies on qRT-PCR machines and reagents. Moreover, integration of this method with robotics liquid handling devices could also help in achieving human-free super high-throughput sample analysis.

A global analysis of incidence of COVID-19 cases across various temperature and humidity zones by Dan *et al.* clearly indicated that variations in humidity and temperature have almost no significance in mortality caused by this virus [30]. In the second week of July 2020, the average daily rise of COVID-19 cases in India is 10–30 thousand cases per day, making it among the top three countries of the world with the highest numbers of infections. This poses a tremendous risk of community infection and a complete disruption of the healthcare system. Countries with high-end healthcare systems such as USA, Russia, Germany, Italy, France, UK, etc., are continuously struggling to curb the infection and reduce number of fatalities. In countries like India where population density is very high, a quick and large-scale sample analysis is very necessary to curb the spread of the virus. Adoption of flow virometry methods will also be useful for scoring community infection studies in metropolitan cities, hospital staffs and the people who are associated with emergency services in this unprecedented time across the globe.

**Executive summary**

- Flow virometry is one of the most important tools for detection of animal viruses and phages.
- Virions of severe acute respiratory syndrome coronavirus-2 could be tagged with specific fluorescent-labeled antibodies.
- Labeled virions could be detected using advanced flow virometry in a high-throughput manner.
- The process requires a biosafety level 3 facility and mechanization such as a robotic arm to minimize droplet infection to the workers.
- The proposed method could enable 2000 tests per day using a single equipment.
